# Scientific Meetings in Medical Oncology: Are We Facing a Time- and Resource-Consuming Plethora?

**DOI:** 10.3390/curroncol33030150

**Published:** 2026-03-05

**Authors:** Vittorio Gebbia, Dario Piazza, Fabrizio Scrima, Alessia Passanisi, Daniela Sambataro, Giuseppa Scandurra, Maria Rosaria Valerio

**Affiliations:** 1 Medical Oncology, Department of Medicine and Surgery, Kore University of Enna, 94100 Enna, Italy; daniela.sambataro@unikore.it (D.S.); giuseppa.scandurra@unikore.it (G.S.); 2 Medical Oncology, Casa di Cura Torina, 90145 Palermo, Italy; dariopiazza@gmail.com; 3 Psychology, Department of Medicine and Surgery, Kore University of Enna, 94100 Enna, Italy; fabrizio.scrima@unikore.it; 4 Department of Human and Social Sciences, Kore University of Enna, 94100 Enna, Italy; alessia.passanisi@unikore.it; 5 Medical Oncology Unit, Ospedale Umberto I, 94100 Enna, Italy; mariarosaria.valerio@unipa.it; 6 Medical Oncology Unit, Ospedale Cannizzaro, 95126 Catania, Italy; 7 Medical Oncology Unit, Policlinico, University of Palermo, 90133 Palermo, Italy

**Keywords:** medical oncology, cancer conferences, meetings, quality

## Abstract

*Background*: In recent years, the rapid advances in molecular biology and cancer pathophysiology, and the rapid availability of new therapeutic agents, have led to an exponential increase in the number of medical oncology conferences. This plethora may partly result from excessive duplication, undertaken without scientific or updating aims, under pressure from sponsors or other motivations unrelated to scientific advancement. The quality of meetings is therefore to be analyzed. *Methods*: A panel of medical oncologists, psychologists, and health-related data managers reviewed the characteristics of 99 out of 125 medical oncology conferences. The meetings were assessed for quality using a 0–5 score based on five parameters: attendees-to-speaker ratio, speaker quality, adequate time allocated for discussion, availability of feedback, and fairness of speeches. *Results*: The panelists identified 25 of 99 scientific events (25%; 95% CI 17–35%) at the 75th percentile and classified them as high-tier meetings, with a total score of 4–5. There were 5 national conferences, 6 regional conferences, and 14 local conferences. Forty-five meetings (56%; 95% Cl 35–56%) reached a score of 0–2 and twenty-nine (29%; 95% Cl 21–39%) a score of 3, and all were considered low tiers. This difference was statistically significant (*p* = 0.002709) in favor of high-titer national conferences. *Conclusions*: Although this paper has several limitations, the results indicate that many conferences were of moderate to poor quality, with a significant prevalence of low-tier events at regional and local levels and a higher concentration of low-tier events within this group. Scientific societies should implement adequate countermeasures.

## Simple Summary

The number of medical oncology conferences has increased exponentially over the last two decades, driven by the rapid evolution of knowledge in molecular biology and cancer pathophysiology, as well as the rapid introduction of numerous new therapeutic agents into clinical practice across a wide range of settings. However, there is concern that many events, especially local and partly regional ones, are of low quality, the result of excessive duplication, and lack scientific and updating aims; instead, they are driven by pressures from sponsors or other motivations unrelated to scientific updating.

## 1. Introduction

Oncology is a branch of medicine characterized by rapid advances in molecular biology and cancer pathophysiology, as well as the rapid introduction of numerous new therapeutic agents into clinical practice across a wide range of settings [1]. Therefore, oncologists must continually strive to stay current with the latest scientific and technological advances [2,3]. The last two decades have seen a significant increase in the number of oncology scientific meetings, especially at the local level [4,5]. The adoption of a multidisciplinary approach to cancer, necessitated by the ever-increasing complexity of the diagnostic and therapeutic pathways, has also contributed to the proliferation of meetings. These events range in size from modest local gatherings, workshops, and symposia to large-scale international congresses that bring together tens of thousands of medical professionals, researchers, exhibitors, and staff for one to four days, often with multiple parallel sessions [4,5]. Attending oncology conferences offers both scientific and professional advantages, including education, professional development, and the exchange of advances and innovations in the field, which may vary with the level of training and experience [6]. However, the impact of conferences and the mechanisms by which effects are generated remain unclear. A qualitative and quantitative analysis conducted by scholars at German universities showed that conferences play a significant role in the qualification process [7]. However, postdocs benefited more than doctoral students from information and networking. In addition to educational value, many conferences offer continuing medical education (CME) credits, providing additional benefit to attendees, particularly in countries that require CME as a prerequisite for practice [8,9]. Benefits can also be psychosocial. In a longitudinal study of emergency physicians, attending conferences was associated with a 3 times lower risk of burnout [10]. 

**Figure 1:**
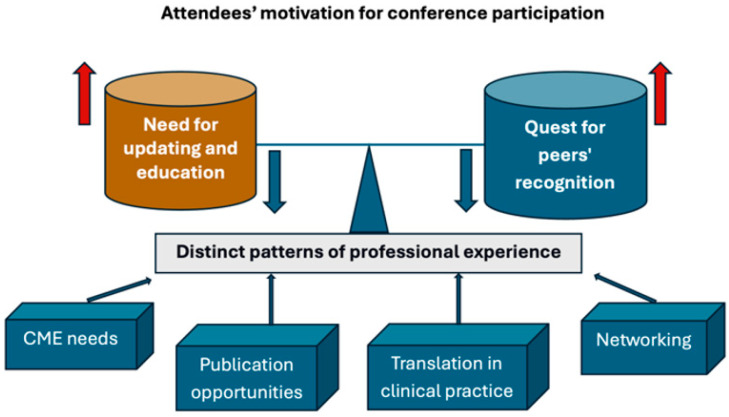
Schematic representation of the reasons behind participation in conferences. Depending on the distinct patterns of professional experience, reasons range from the need for continuing medical education and translation into clinical practice to opportunities for publication and networking.

Figure 1 presents a schematic representation of the reasons for participating in conferences. These meetings also provide an opportunity to establish or reinvigorate scientific and professional networks [4,6,7]. In fact, such meetings are social settings in which oncologists meet colleagues, build networks, and engage in exploratory conversations, in addition to serving as a means of scientific growth [7]. An analysis of a database of 270 in-person national and international academic conferences held between 2018 and 2019 revealed that, in many meetings, there was room for significant improvement in diversity and inclusivity, as well as in promoting early-career researcher networking, career development, and venue accessibility [11]. While this phenomenon can be attributed to the constant, rapid, and ever-growing progress in many neoplasms and clinical settings, the sheer number of meetings, often duplicated, raises questions about their quality, necessity, and the motivations behind this proliferation of local events [4,5,12]. A scoping review mapping the published conference evaluation literature (83 studies from 2008 to 2022) across multiple medical fields failed to identify a validated tool for conference evaluation, suggesting that organizers and research teams are developing their own instruments [12]. The authors also conducted a content analysis to identify the relevant domains for conference evaluation, suggesting four primary domains (engagement and networking, education and learning, impact, and scholarship) and an additional four domains identified through the content analysis (value and satisfaction, logistics, equity, diversity, and inclusivity, and career influences). 

Although a shared measure of conference quality has not been established to date, the organizers’ reputation and history play a crucial role in ensuring the conference’s quality. Unquestionably, sound organizations such as the American Society of Clinical Oncology (ASCO), the American Association for Cancer Research (AACR), the European Society of Medical Oncology (ESMO), American Society for radiation Oncology (ASTRO), and the San Antonio Breast Cancer Conference, etc., in Western countries, assure quality, prestige, longevity, popularity, and periodicity. These large conferences typically present concurrent section programs featuring high-quality speakers who cover both educational and cutting-edge topics, and they provide either paper or digital proceedings. Bibliographic citations of published abstracts or lectures are pivotal in elaborating quality metrics [13,14]. The above-mentioned outstanding institutions ensure transparency in peer review, independent of acceptance rates that may be influenced by non-scientific factors. On the other hand, the coverage of low-tier conferences is generally extensive, spanning multiple fields. In comparison, high-tier conferences typically focus on a single area, subject, or theme, or present a range of parallel sessions. 

Unfortunately, as in all human activities, oncology conferences may also present less transparent aspects, or even darker ones, such as predatory meetings, a scarcely studied phenomenon with only one paper exploring the reasons for participation in such settings [4,15]. A scoping review of predatory conferences revealed that they often rely on misleading information and attractive locations, underscoring the need for countermeasures that emphasize the responsibilities of universities and funders, as well as the publication of lists of predatory publishers associated with conferences [15]. The reasons for the duplication and multiplicity of conferences, especially small ones, may be non-scientific. Hot and controversial points, often challenging to interpret unequivocally, may include personal career reasons, visibility, industry interests, profit, and other factors, frequently mixed in variable proportions [16]. Some authors have feared that meetings may also establish a branding strategy that enhances scientists’ standing in the field and encourages following prominent opinion leaders, whose important statements may have a significant impact, even in the absence of irrefutable proof. Although there is little guarantee that the views of these highlighted speakers are meritorious or of high quality, securing the podium for a plenary address or key sessions at a major convention confers prestige [4]. These considerations have sparked considerable debate in the medical literature, with both pro and con arguments [4,17,18,19,20]. From a psychological perspective, participation in scientific meetings can be conceptualized as a motivated behavior shaped by basic psychological needs for autonomy, competence, and relatedness, as described by self-determination theory [21]. These needs are fulfilled when professionals perceive conferences as contexts where they can freely choose high-quality learning opportunities, feel effective in updating their knowledge, and experience meaningful connections with colleagues. Social identity approaches further underline that oncologists derive part of their professional self-concept from belonging to valued expert communities and prestigious societies [22]. In this view, conferences do not only transmit information, but also function as rituals that confirm status, identity, and recognition within the oncology community. When events are well designed, they can satisfy these needs and foster intrinsic motivation, engagement, and well-being; conversely, highly commercial, repetitive, or low-quality meetings risk thwarting these needs and may contribute to cynicism and burnout [23]. Moreover, psychological and sociological research shows that “weak-tie” relationships formed in such settings are a key source of novel information, opportunities, and social capital, which can indirectly influence career development and scientific collaboration [24]. Integrating these psychological dimensions is therefore essential to evaluate the real impact of the current plethora of oncology meetings on professionals’ motivation and mental health.

In this paper, we present an analysis of the characteristics and quality of scientific meetings held in southern Italy over the last decade. The present analysis identified some of the pitfalls, if any, of oncology conferences suggesting possible organizational and policy-related improvements and ultimately benefiting healthcare professionals, researchers, patients, and the community.

## 2. Materials and Methods

### 2.1. Data Analyzed

The analysis included 125 scientific events on medical oncology and its multidisciplinary aspects held in southern Italy, from January 2015 to November 2025. The study included both in-person and virtual meetings. Events were defined as follows: (a) conferences, usually large international or national multi-track events, often held annually, comprising plenary and poster sections and a preplanned proceeding publication; (b) round table, small, informal discussions for a group to exchange ideas on a specific issue; seminars, educational sessions with presentations followed by a question-and-answer period or discussion; (c) workshops, hands-on sessions designed for practical, skill-based learning in a specific subject; (d) panel discussion, moderated conversation where multiple experts discuss a topic, often including audience participation through questions. The authors evaluated events lasting 1 to 3 days, including large conferences, workshops, seminars, and symposia.

### 2.2. Rules for Event Organization

In Italy, among numerous other healthcare aspects, the Government Agency for Health Services (AGENAS, Agenzia Nazionale per i Servizi Sanitari Regionali) oversees CME and supervises and approves conferences [9]. Any organization or provider needs the agency’s approval and accreditation to organize a congress at any level and to meet specific requirements regarding the organization and quality of the training offered. The agency rules require healthcare professionals to obtain ECM credits, comply with event accreditation requirements (e.g., completing a satisfaction questionnaire), and meet specifications for attendee participation (e.g., attending at least 90% of the event and passing the learning assessment) [9]. Product data or information sheets may not be presented in a way that interferes with the scientific program; only the brand name is permitted. In the case of sponsorship, the provider must inform participants before the event begins and declare the presence of sponsors. Participants must complete the evaluation form to assess the event’s perceived quality and, if applicable, undergo a final learning test.

### 2.3. Event Evaluation

A panel of seven experts, including four medical academic oncologists, two academic medical psychologists, one data manager, and one experienced health-related data manager, reviewed all events. The type of events included national, regional, and local conferences. National conferences involved all national oncologists. Regional meetings included oncologists from an entire geographic/political region and were not repeated throughout the year. Local meetings involved oncologists from a city or province. The study considered the following parameters: (1) sponsorship (institutions versus industry versus both); (2) relevance of speakers as evaluated by H-index (low versus high), scientific papers production in specific fields as accessed on PubMed and Scopus and/or proven clinical activity volume; (3) time for open discussion after each session (insufficient <10 min; suboptimal 10–20 min >20 min as adequate); (4) monothematic versus polythematic events; (5) type of topic: drug-focused versus strategy oriented ones; (6) number of speakers/number of attendees ratio; the closer the speakers/attendee ratio is to 1, the smaller the number of participants, while the smaller is the ratio, the greater the number of participants compared to the number of speakers. This data gives information on the number of speakers and attendees per conference. A high ratio indicates a lack of interest in the meeting, while a low ratio indicates greater participation. (7) presence of structured checklists and published attendees’ feedback evaluated by open interview. Panelists also evaluated duplication of meetings.

### 2.4. Quality of Conferences

The expert panel assessed the quality of all conferences using a 0 to 5 score based on five parameters: 1. attendees/speaker ratio (low ratio <1:2/high ratio ≥1:2), which measured interest in the theme/event; quality of speakers (low/high), based on publications (no or less than 2 publications on indexed journal) as a measure of specific knowledge, and H-index for global scientific assessment; adequate time allocated for discussion (yes, ≥15 min/no, <15 min), as a measure of room for opinion and data exchange; availability of feedback (yes/no), as an indicator of educational value and satisfaction; and fairness of speeches (>61 Cohen’s Kappa coefficient to reduce the selection biases and achieve a substantial to almost complete agreement between researchers), as a measure of the objectivity of the data presented. Panelists converted all scoring data into percentiles. Conferences in the top 75th percentile were considered high-tier, and the other were considered low-tier.

### 2.5. Statistics

Descriptive statistics included absolute numbers with their relative percentages. Percentages were rounded to the nearest unit, and their 95% confidence limits (95%Cl) were reported. A chi-square test was applied to a contingency table for comparative analysis. *p*-values for chi-square and correlation statistics were reported at the 0.05 significance level. Statistical correlation analysis and graph generation were performed using GraphPad Prism version 10.1.0 (GraphPad Software, Boston, MA, USA).

## 3. Results

Although not required by Italian Government regulations for medical studies, the project was approved by the Ethical Committee of Kore University of Enna, Enna, Italy. Of the 125 scientific events reviewed, 27 (22%) were excluded from the analysis due to insufficient cooperation from some providers, resulting in missing data for evaluation. Most of the excluded events were local. Excluded conferences were 19 local (70%), 8 regional (30%), and 5 national (5%) events. All but five of the events did not include prearranged publication of proceedings in indexed journals. Only the proceedings from the five national congresses were published in a highly indexed journal.

Table 1 shows the main characteristics of the 95 events included in the final analysis. The large national congresses of the Italian Association of Medical Oncology (AIOM) were excluded because they met all criteria for a top-tier conference. Overall, nearly half of the meetings were monothematic; however, 79% of the regional conferences were polythematic, whereas 61% of the local ones were monothematic. This difference was statistically significant (*p* = 0.00001). Pharmaceutical companies, either directly or in partnership with other institutions, sponsored 97% of all meetings. Sixty-three percent of meetings lasted one day. However, a statistically significant difference in duration was observed among national, regional, and local meetings (*p* = 0.005165), with regional meetings lasting longer than local ones. The median number of attendees was lower at local conferences than at regional conferences (25 versus 72), whereas the median number of speakers was higher at regional events than at local events (28 versus 14). The speakers-to-attendees ratio was much lower in the national congresses. The index for regional meetings was lower than that for local meetings (0.38 versus 0.46). Adequate time for discussion was suboptimal in 70% of meetings, except for national ones (*p* = 0.01892). Feedback to the organizer and attendees, or written output, was absent in 90% of cases (*p* < 0.00001). Only national meetings published proceedings in indexed journals. The quality of speakers, as evaluated by H-index and indexed publications, was suboptimal in 29% and 40% of regional and local meetings, respectively. The volume of clinical activity among speakers was low in 12% of regional and 43% of local meetings, respectively (*p* = 0.001116). In 5 events, some of the speakers had no known clinical activity. Duplication was statistically higher in local than in regional or national meetings (*p* = 0.005646).

**Figure 2:**
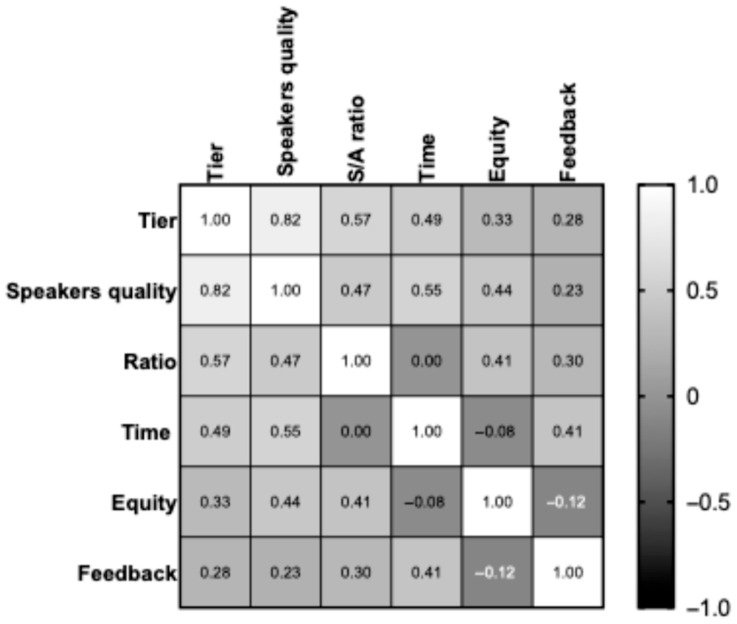
Heath map showing the correlation between tiers and quality scoring parameters.

As shown in Table 2, the panelists identified 25 of 99 scientific events (25%; 95% CI 17–35%) at the 75th percentile and classified them as high-tier meetings, with a total score of 4–5. There were 5 national conferences, 6 regional conferences, and 14 local conferences. Forty-five meetings (56%; 95%Cl 35–56%) reached a score of 0–2, and twenty-nine (29%; 95%Cl 21–39%) a score of 3, and all were considered low tiers. This difference was statistically significant (*p* = 0.002709) in favor of high-titer national conferences. High-tier meetings corresponded to the first percentile of all data. Figure 2 illustrates the heath map, showing the correlation between tiers and quality-scoring parameters. The correlation between tiers and speakers’ quality, speakers-to-attendees ratio, time for discussion, equity of speeches, and feedback availability was statistically significant (Pearson’s r ≤ 1).

## 4. Discussion

In oncology, scientific conferences play a pivotal role in divulging knowledge, enhancing collaboration, and disseminating cutting-edge research [1,2,3,6,25]. To improve the quality of health care, clinical conferences enable scientists, researchers, and healthcare professionals to engage directly and examine current issues within a specific clinical context. Challenges such as high costs, geographic limitations, and the need for sustainable practices can be addressed by adopting hybrid and virtual formats, making conferences more accessible and inclusive [25].

Despite the advantages mentioned above, the number of meetings per geographical area may be too high, and the quality of cancer conferences may be suboptimal [3,5]. In fact, the need to improve the quality of cancer conferences is not a new issue [26]. Nearly thirty years ago, researchers at the University of Illinois College of Medicine in Chicago, USA, raised concerns about quality, which others echoed almost every ten years. The latter emphasized issues of redundancy and the duplication of meetings, as well as the risks linked to this abundance [4,5,26].

General psychology and occupational health psychology offer additional lenses through which to interpret our findings. Models of burnout emphasize that chronic exposure to high job demands in combination with low resources erodes meaning, produces emotional exhaustion, and fosters depersonalization [23]. When oncologists repeatedly participate in conferences that are highly duplicative, densely scheduled, and strongly influenced by commercial interests, but that offer limited opportunities for genuine discussion, feedback, or skills development, these events may function as an additional stressor rather than a protective resource. In terms of self-determination theory, such meetings are likely to provide little autonomy, competence, or relatedness support, thereby increasing the risk of controlled forms of motivation and burnout rather than high-quality motivation [21]. At the same time, cultures that implicitly reward constant visibility at meetings and uninterrupted productivity can exacerbate impostor feelings and self-doubt among clinicians and researchers, particularly those at earlier career stages [27]. Recent psychological work shows that repeated rejection, impostor syndrome, and burnout are widespread in academic and clinical environments, and that structural expectations and norms play a central role in sustaining these experiences [27]. A psychologically informed assessment of conference quality should therefore consider not only structural indicators (such as speakers’ H-index or proceedings publication), but also how far events support or undermine professionals’ basic psychological needs, social identity, and long-term well-being.

Evaluating the quality of a scientific conference is a complex and multifaceted issue [28]. It should rely on a combination of criteria rather than a single factor. In the medical literature, several papers suggest rules, checklists, and formulas for organizing a scientific meeting [29,30,31]. The goal is to provide a quantitative measure that goes beyond subjective assessments, helping attendees make informed decisions about which sessions to attend, particularly regarding the time, energy, and financial resources they allocate. The influence of the meeting’s conclusions, the significance of the presentations, and the standing of the organizers are among the theoretical methods used to evaluate the quality of scientific gatherings. Generally, high attendance levels are considered a quality indicator, but the number of attendees may not necessarily reflect the scientific quality.

**Figure 3:**
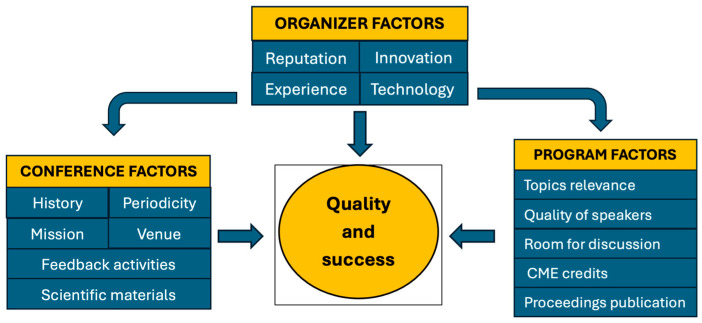
Factors contributing to the quality and success of cancer conferences. Factors are divided into four groups: conference-, organizer-, and program-related factors, which are interrelated in various ways.

On the other hand, the organizer’s reputation and experience are key factors, based on their demonstrated commitment to quality and scientific rigor, as well as on the conference’s history, mission, popularity, prestige, past events, and periodicity [13]. The relevance of topics and the presence of prominent speakers are indicators of the conference’s significance and quality. Based on research data and their personal observations, speakers should identify the most pressing problems, evaluate the findings, and propose possible solutions for attending health professionals [6]. Many papers report publication opportunities as a hallmark of high-quality conferences, often with proceedings published in reputable journals or indexed in databases. Figure 3 illustrates the factors that contribute to the quality and success of cancer conferences.

Today, however, there is no universally accepted set of metrics for assessing conference quality. By enforcing strict peer-review processes for ideas and presentations, cancer conferences maintain high scientific standards and ensure the applicability and relevance of the topics. State-of-the-art research, comprehensive data, and a reliable methodology are essential for effectively illustrating scientific progress. Effective time management, schedule adherence, and opportunities for one-on-one or small-group interactions are examples of organizational efficiency metrics that may be relevant. Innovative formats can enhance conference participation and learning while also providing logistical and technological support. They can also facilitate post-meeting activities or actions.

On the other hand, high attendance levels are considered a quality indicator, but the number of attendees may not necessarily reflect the scientific quality. However, there is no universally accepted set of metrics for measuring the quality of conferences. The quality of scientific meetings held by outstanding institutions relies on a rigorous peer review process, expert speakers and panelists, and presentations based on substantial evidence. Generally, these conference plans include publication of the proceedings in a prearranged manner. In this case, the editorial committee of recognized experts will employ a peer-review process, utilizing the abstract publication rate and impact factor of the journals where abstracts are published, as these are standard metrics for assessing journal quality.

Several studies have examined the quality of scientific meetings, their impact on attendees, and methods for evaluating these aspects [32]. Sufi et al. provided a set of recommendations for measuring the effect of workshops, focusing on planning, updated knowledge, speakers’ skills, and feedback [32]. An international panel also published a step-by-step guide for organizing the scientific program of international conferences, which includes a flowchart, a checklist, and detailed discussions of the inputs and outputs for each step [33]. This guide outlines six steps: (1) preparation, (2) recruitment, (3) building the agenda, (4) cross-checking the program, (5) reviewing and finalizing, and (6) in-conference refinement. Thirteen items are detailed across these six main steps in a comprehensive checklist. Altan et al. evaluated the quality of scientific meetings of the Turkish Society of Urological Surgery based on the impact factor of the journals in which the abstracts were published, as well as the H-index of the scientists invited as speakers to the congress, using the Web of Science database [28]. The authors employed a quality factor (QF) measure calculated as follows: [(abstract publication rate over 2 years × average journal impact factor) + average H-index of speakers]/10. H-index may reflect the quality and competence of the speakers. H-index and recognized expertise are common indicators of a speaker’s quality, even if they do not necessarily imply strong communication skills [34]. The authors acknowledged the need for further refinement and standardization of the formula, emphasizing the importance of a standardized calculation tool to objectively assess meeting quality.

Although criteria for measuring conference scientific output have been proposed, achieving this goal remains challenging [35,36,37]. Zhuang et al. introduced a set of innovative heuristics to automatically identify prestigious or low-quality conferences by analyzing the characteristics of program committee members [37]. They accurately classified about 92% of nearly 3000 meetings, identified almost 16,000 committee members, and maintained a low false-positive rate of 0.035. Their approach also effectively detected low-quality conferences, with a false-positive rate of just 0.002. However, citations do not help evaluate smaller, local meetings that do not publish conference proceedings. This pattern is typical in small local meetings, often due to limited funding, lack of novelty, and the quality of speakers.

The internal dynamics of conference participation and their contributions to learning processes in communities of health professional practice remain poorly studied. Indeed, the motivating environment of medical conference attendees is somewhat complex, with the pursuit of peer recognition often balancing the need for education [38]. A qualitative interview- and observation-based study analyzed the roles of the physical environment, participant motivation, interdisciplinarity, and attendee diversity [38]. The study identified four categories of congress attendees: explorers, newcomers, drivers, and updaters. Distinct patterns of professional experience and involvement in the professional communities distinguished each group. Active engagement on social media platforms can reflect a conference’s relevance and community involvement. Harnessing gamification to test participants’ skills may be helpful. Moreover, by uploading all conference presentations, posters, and abstracts to popular public repositories for each category of information, the impact of scientific conferences may be increased [39]. The adoption of innovative conference technologies adds value to the scientific event, potentially expanding access, engagement, and diffusion. For instance, collaborative platforms may facilitate effective networking and engagement in discussions with peers; however, they do not guarantee the quality of the interactions themselves. Summaries, recordings, slide decks, and other materials from post-conference sessions can serve as valuable resources for enhancing feedback and knowledge sharing. Congress-organizing agencies should collect feedback from attendees and sponsors to assess the conference’s efficacy and success and use it to inform improvements for future events. However, such results are usually not discussed with the scientific committee that prepared the agenda, and success is often superficially assessed based on the number of participants or on randomly collected verbal opinions in a non-scientific manner.

Our study found that many conferences were of moderate to poor quality, with a high prevalence of low-tier events at the regional and local levels, and a higher concentration of such events within this group. The exclusion of 22% of the scientific events was related to non-cooperative attitude of some scientific event providers and/or incomplete data provision for instance the number of actual participants both among the audience and the speakers, or the provision of grants to chairmen, speakers or moderators. High-tier conferences had a speakers-to-attendees ratio considerably lower than that of low-tier events, indicating higher demand and quality. Pharmaceutical companies organized more than 50% of the meetings directly and sponsored nearly all included in the analysis, including those organized by cooperative groups, scientific associations, and other entities. Duplication was higher in local than in regional or national meetings, supporting the hypothesis that factors other than scientific updating are at play. However, some industry-driven meetings are oriented toward informing oncologists about the optimal management of new agents or combination therapy. The average quality of speakers was suboptimal, as in 29–40% of cases they had not published in the field or had no significant scientific output, based on the number of published papers in the field. In numerous instances, the conference structure was inadequate, mainly due to a polythematic program with limited interactive discussion and a too-narrow agenda, resulting in significant schedule delays and creating a vicious cycle. Several meetings, primarily those driven by industry, seemed more oriented to marketing than to science. In contrast, other meetings proposed by oncologists and sponsored by industry appeared to have self-promotion purposes rather than scientific updating objectives. These findings can be interpreted through the lens of the Impression Management Theory, which suggests that, particularly at the local and regional levels, conferences may be organized not primarily for scientific updating but rather to enhance one’s visibility within the professional community and to strengthen personal reputation [40]. Furthermore, additional insights may be provided by the Social Identity Theory and, more specifically, by the subsequent Self-Categorization Theory, which posit that the organization of smaller conferences at the local or regional level may ultimately serve to reinforce group identity and foster a sense of belonging, even at the expense of scientific quality [41].

Our work, however, has several limitations. First, the geographical area in which the meetings took place may not reflect the situation across the entire nation or in other countries. However, there is some consistency, at least in the Western world, on this subject. A second limitation concerns the extrapolation of data from large-scale meetings, at least at the national level, which typically publish abstracts and proceedings. Therefore, the reported data apply only to smaller regional and local meetings. On the other hand, the inclusion of national meetings in the analysis may represent another point, since large nationwide conferences are usually organized by reputable scientific associations with recognized scientific boards. This latter data may help highlight the pitfalls of small meetings. Finally, the absence of data on participants’ feedback prevents a more accurate assessment of the scientific and educational impact on attendees.

Because some conferences may influence oncologists’ prescribing practices or even healthcare policies and guidelines, sponsorship is a sensitive issue, as it can raise concerns about potential biases or conflicts of interest [42,43]. Ministries and Departments of Health typically organize these conferences with leading research and/or educational institutions in the field, recognized medical centers, and other health-related organizations. However, pharmaceutical companies are heavily involved in medical events, sponsoring many, if not most, scientific conferences and congresses. Despite this support for knowledge dissemination, such involvement poses a threat to the mission of these events [16]. 

In fact, the role of pharmaceutical companies in CME or cancer conferences can be complex, like Plato’s myth of the chariot in the Phaedrus, where the white and black horses represent good and evil, respectively, and human behavior results from these conflicting forces. Partnerships between academic oncology and industry are crucial for advancing cancer treatment. However, there has been limited research on engagement with and perceptions of these relationships [44]. An electronic survey of 225 members of the American Society of Clinical Oncology, mostly US-based medical oncologists, examined the connections between academic oncology and industry. Overall, 86% reported an active relationship with the industry and viewed such collaborations as important for their careers. Most respondents (92%) believed that scientific integrity was upheld, and 95% reported that the quality of collaborative work was rarely compromised. However, 60% expressed concerns about potential conflicts of interest related to industry funding for clinical care and research. Despite these concerns, the majority (67%) stated that these relationships did not influence their interactions with patients. 

Corporate interests can shift research priorities away from topics most relevant to public health. This issue raises some unsettling ethical questions about who oversees the creation of conference agendas, for whom medical conferences are held, and whether these events genuinely improve health or merely support evidence-based medicine [45,46]. Unfortunately, conferences tend to proliferate, as different departments and agencies host similar events, often featuring presentations that are frequently prearranged by pharmaceutical companies and that lack clear scientific innovation. Meanwhile, these conferences have mainly become platforms for advertising new technologies or pharmaceuticals [45,46]. Pharmaceutical companies invite speakers and attendees, providing extensive financial support and honoraria, with amounts varying based on the level of importance. A scoping review summarized 36 studies examining the influence of industry sponsorship on research agendas across various medical fields [47]. Seven of these studies analyzed internal industry records and uncovered tactics used to reshape entire areas of research, focusing on topics aligned with industry legal and policy positions. According to 10 studies that employed questionnaires and interviews to explore researchers’ experiences and perspectives, researchers were primarily aware that sponsorship could influence their research goals.

Direct payments from the pharmaceutical industry to U.S. physicians are common, have increased over time, and may influence physicians’ clinical practice and interpretation of clinical trial results [48]. The increasing prevalence and complexity of relationships between industry and oncologists raise ethical challenges [45]. They also constantly navigate changing conflict-of-interest policies within academic centers and professional societies [48]. A total of 13,087 medical oncologists received payments. The average annual payment per physician increased from $3811 in 2014 to $5854 in 2017. Oncologists who served on NCCN Guidelines Panels received higher payments and experienced a greater relative increase, with average payments rising from $10,820 in 2014 to $18,977 in 2017, particularly in the field of immunotherapy [49]. As foreseen by Dr. M. Ratain in 2015, in the USA, industry payments to physicians and oncologists are now publicly searchable under the Physician Payments Sunshine Act because financial incentives unrelated to research may influence oncology care since transparency and scrutiny are essential to monitor and reduce potential conflicts of interest for ethical reasons [48,49,50,51,52,53]. For example, pharmaceutical companies paid nearly $2 million to 765 gynecologic oncologists, with 48 receiving $10,000 or more from the industry. Professional organizations, including the Society of Gynecologic Oncology (SGO), should proactively develop guidelines on interactions with industry for their members [53]. Among medical or hematology-oncologists, payment patterns exhibit seasonal peaks and increase significantly during major oncology conferences [51]. Strategies to counteract corporate influence on the research agenda are needed, including the heightened disclosure of funding sources and conflicts of interest in published articles, to facilitate an assessment of commercial biases. Other recommendations to improve academic-industry partnerships may include a scientific societies-certified checklist in planning scientific events. The events should include speakers and discussants of proven scientific experience which may be lacking especially in small local events to avoid misinterpretation of data, a greater fostering activity and involvement of scientific societies which often is confined to the granting of the logo on the event poster or the scientific aegis. of discussions. Especially in the case of industry-organized events, the selection of speakers should not be influenced by the actual volume of prescriptions or known opinions to assure equity and pluralism.

## 5. Conclusions

In conclusion, our study shows that, with some exceptions, the overall quality of local cancer conferences is generally poor. The main issue is the poor quality of speakers, who often have little to no scientific background or clinical experience, along with a tendency toward duplication and other problems, such as excessive and unbalanced commercial or self-promoting aims. Scientific societies, which are often overly prone to granting their patronage to scientific events of little relevance, should pay closer attention and oversee the proposed programs. Although industry support for academic and research institutions remains pivotal, guidelines for industry support of educational conferences should be improved. Finally, the usefulness and appeal of in-person conferences, especially small and local ones, are challenged by the pervasive use of artificial intelligence [21,22,54,55].

## Figures and Tables

**Table 1:** Characteristics of examined cancer conferences.

All			Scope			
			National	Regional ^	Local ^^	
N. of conferences	99 (100%)		5 (100%)	24 (100%)	70 (100%)
Themes	Monothematic	48 (48%)	0	5 (21%)	43 (61%)	*p* = 0.00001
	Polythematic	45 (45%)	5 (100%)	19 (79%)	27 (39%)	
	Parallel sections	5 (5%)	5 (100%)	0	0	
Sponsors	Institutions	3 (3%)	0	0	3 (4%)	*p* = 0.400602
	Industry	50 (51%)	0	13 (54%)	37 (53%)	
	Both	46 (46%)	5 (100%)	11 (46%)	30 (43%)	
Length	1 day	62 (63%)	0	9 (37%)	53 (76%)	*p=* 0.005165
	2 days	18 (18%)	0	7 (29%)	11 (16%)	
	3 days	19 (19%)	5 (100%)	8 (33%)	6 (8%)	
Attendees Number	Median	Not applicable	3000	72	25	
	Range		na *	44–128	15–78	
Speakers’ number	Median Range	Not applicable	295 na *	28 20–45	14 5–25	
Speaker/Attendee ratio		Not applicable	0.098	0.38	0.56	
Speakers’ quality **	High	64 (66%)	5 (100%)	17 (71%)	42 (60%)	*p* = 0.377968
	Low	35 (44%)	0	7 (29%)	28 (40%)	
Speakers’ volume of clinical activity	High	59 (60%)	5 (100%)	21 (87%)	33 (47%)	*p =* 0.001116
	Low	40 (40%)	0	3 (12%)	37 (43%)	
Time for discussion	Adequate	32 (33%)	4 (80%)	6 (25%)	21 (30%)	*p =* 0.01892
	Inadequate	66 (67%)	1 (20%)	18 (75%)	49 (70%)	
Proceedings	Yes	5 (5%)	5 (100%)	0	0	
	No	94 (95%)	0	0	0	
Written output	Yes	11 (11%)	5 (100%)	1 (4%)	5 (7%)	*p* < 0.00001
	No	88 (89%)	0	23 (96%)	65 (93%)	
Feedback to attendees	Yes	14 (14%)	5 (100%)	2 (8%)	7 (10%)	*p* < 0.00001
	No	85 (86%)	0	22 (92%)	63 (90%)	
Duplication	Yes	29 (29%)	0	2 (8%)	27 (39%)	*p =* 0.005646
	No		5 (100%)	24 (92%)	43 (61%)	

^ Regional: meeting involving an entire geographic/political region, not repeated during the year; ^^ Local meetings were those involving oncologists from a city or a small province; * na: Not available; ** As evaluated by H-index and publications.

**Table 2:** Conferences are tiered according to the panelist’s score.

Evaluation		Number of Medical Oncology Conferences (Total 99)			
		National (n. 5)	Regional (n. 24)	Local (n. 70)	All (n. 99)
Score	4–5	5	6 (25%)	14 (20%)	25 (25%)
	3	0	8 (33%)	21 (30%)	29 (29%)
	0–2	0	10 (42%)	35 (50%)	45 (45%)
Tier *	High	5 (100%)	6 (25%)	14 (20%)	25 (25%)
	Low	0	18 (75%)	56 (80%)	74 (75%)

* The *p*-value is 0.002709.

## Data Availability

The data presented in this study are available from the corresponding author upon request, subject to the following restriction: involvement of third parties who do not directly participate in the study.

## Abbreviations

The following abbreviations are used in this manuscript:

CME	Continuous Medical Education

## References

[1] Liu B., Zhou H., Tan L., Siu K.T.H., Guan X.Y. (2024). Exploring treatment options in cancer: Tumor treatment strategies. Signal Transduct. Target. Ther..

[2] Beatty P.A. (2012). Coping with abundance: The burden of progress in medical oncology. Oncologist.

[3] Schmidinger M., Bellmunt J. (2010). Plethora of agents, plethora of targets, plethora of side effects in metastatic renal cell carcinoma. Cancer Treat. Rev..

[4] Ioannidis J.P. (2012). Are medical conferences useful? And for whom?. JAMA.

[5] Al Hadidi S. Too Many Oncology Conferences: What Is Feasible?. https://connection.asco.org/do/too-many-oncology-conferences-what-feasible.

[6] Sciortino F. (2018). Why organizing a scientific conference can produce huge benefits. Nature.

[7] Hauss K. (2020). What are the social and scientific benefits of participating at academic conferences? Insights from a survey among doctoral students and postdocs in Germany. Res. Eval..

[8] Continuing Education & Maintenance of Certification. https://www.asco.org/meetings-education/continuing-education-moc.

[9] Educazione Continua in Medicina. https://www.agenas.gov.it/aree-tematiche/formazione-e-supporto-al-programma-nazionale-ecm/educazione-continua-in-medicina-ecm.

[10] Cydulka R.K., Korte R. (2008). Career satisfaction in emergency medicine: The ABEM longitudinal study of emergency physicians. Ann. Emerg. Med..

[11] Sarabipour S., Khan A., Seah Y.F.S., Mwakilili A.D., Mumoki F.N., Sáez P.J., Schwessinger B., Debat H.J., Mestrovic T. (2021). Changing scientific meetings for the better. Nat. Hum. Behav..

[12] Albrecht L., Pratt M., Ng R., Olivier J., Sampson M., Fahey N., Gibson J., Lobos A.T., O’Hearn K., Newhook D. (2024). Measuring continuing medical education conference impact and attendee experience: A scoping review. Int. J. Med. Educ..

[13] Martins W.S., Goncalves M.A., Laender A.H.F., Ziviavi N. (2010). Assessing the quality of scientific conferences based on bibliographic citations. Scientometrics.

[14] Vahdati S., Fathalla S., Lange C., Behrend A., Say A., Say Z., Auer S. (2021). A comprehensive quality assessment framework for scientific events. Scientometrics.

[15] Godskesen T., Eriksson S., Oermann M.H., Gabrielsson S. (2022). Predatory conferences: A systematic scoping review. BMJ Open.

[16] Legg T., Hatchard J., Gilmore A.B. (2021). The Science for Profit Model-How and why corporations influence science and the use of science in policy and practice. PLoS ONE.

[17] Cumberworth A., Cumberworth J., Sharp S. (2012). Usefulness of medical conferences. JAMA.

[18] Antman E.M., Harrington R., Tomaselli G. (2012). Usefulness of medical conferences. JAMA.

[19] van Meurs M., Dijkema L.M., Zijlstra J.G. (2012). Usefulness of medical conferences. JAMA.

[20] Braun M. (2012). Usefulness of medical conferences. JAMA.

[21] Ryan R.M., Deci E.L. (2000). Self-determination theory and the facilitation of intrinsic motivation, social development, and well-being. Am. Psychol..

[22] Tajfel H., Turner J.C., Austin W.G., Worchel S. (1979). An Integrative Theory of Intergroup Conflict.

[23] Maslach C., Leiter M.P. (2016). Understanding the burnout experience: Recent research and its implications for psychiatry. World Psychiatry.

[24] Granovetter M.S. (1973). The strength of weak ties. Am. J. Sociol..

[25] Guetter C.R., Altieri M.S., Henry M.C.W., Shaughnessy E.A., Tasnim S., Yu Y.R., Tan S.A. (2022). In-person vs. virtual conferences: Lessons learned and how to take advantage of the best of both worlds. Am. J. Surg..

[26] Guinan P.D., Imperato J.P., Chmiel J.S., Vogelzang N.J., Sylvester J. (1997). Cancer conferences. Can they be improved?. Cancer Pract..

[27] Jaremka L.M., Ackerman J.M., Gawronski B., Rule N.O., Sweeny K., Tropp L.R., Metz M.A., Molina L., Ryan W.S., Vick S.B. (2020). Common academic experiences no one talks about: Repeated rejection, impostor syndrome, and burnout. Perspect. Psychol. Sci..

[28] Altan M., Ayva M., Bahadır Ö.F., Kısıklı A., Baltacı K.E., Shahsuvarlı P., Bozacı A.C., Doğan H.S., Tekgül S. (2025). An Objective Quantitative “Quality Factor” for Scientific Meetings, Is It Possible? A New Formula. J. Urol. Surg..

[29] Corpas M., Gehlenborg N., Janga S.C., Bourne P.E. (2008). Ten simple rules for organizing a scientific meeting. PLoS Comput. Biol..

[30] Chicco D., Bourne P.E. (2022). Ten simple rules for organizing a special session at a scientific conference. PLoS Comput. Biol..

[31] McInerny G.J. (2016). Ten Simple Rules for Curating and Facilitating Small Workshops. PLoS Comput. Biol..

[32] Sufi S., Nenadic A., Silva R., Duckles B., Simera I., de Beyer J.A., Struthers C., Nurmikko-Fuller T., Bellis L., Miah W. (2018). Ten simple rules for measuring the impact of workshops. PLoS Comput. Biol..

[33] Aljazeeri I., Lorens A., Offeciers E., Saleh E., Mertens G., Skarzynski H., Alrand H., Anderson I., Mueller J., Van de Heyning P. (2023). A Good Practice Guide for Organizing the Scientific Program of International Conferences. Cureus.

[34] Hnatiienko H., Snytyuk V., Tmienova N. Calculation of the integral quality index of a scientific event in the context of the interests of a scientific institution. Proceedings of the XXI International Scientific and Practical Conference “Information Technologies and Security” (ITS-2021).

[35] Kreiman G., Maunsell J.H. (2011). Nine criteria for a measure of scientific output. Front. Comput. Neurosci..

[36] Fadlelmola F.M., Panji S., Ahmed A.E., Ghouila A., Akurugu W.A., Domelevo Entfellner J.B., Souiai O., Mulder N. (2019). H3ABioNet Research working group as members of the H3Africa Consortium. Ten simple rules for organizing a webinar series. PLoS Comput. Biol..

[37] Zhuang Z., Elmacioglu E., Lee Giles C.L. Measuring Conference Quality by Mining ProgramCommittee Characteristics. Proceedings of the 7th ACM/IEEE Joint Conference on Digital Libraries.

[38] Schneider-Kamp A., Petersen F. (2025). Made for or Made by? A Qualitative Investigation into the Diverse Practices and Roles of Medical Congress Participants. Qual. Health Res..

[39] Morrison M., Merlo K., Woessner Z. (2020). How to Boost the Impact of Scientific Conferences. Cell.

[40] Buckwell C. (2008). Should the drug industry work with key opinion leaders? Yes. BMJ.

[41] Fava G.A. (2008). Should the drug industry work with key opinion leaders? No. BMJ.

[42] Harrison R.A., Majd N.K., Johnson M.O., Urbauer D.L., Puduvalli V., Khasraw M. (2023). Characterization of industry relationships in oncology. Cancer.

[43] Robertson C., Rose S., Kesselheim A.S. (2012). Effect of financial relationships on the behaviors of health care professionals: A review of the evidence. J. Law. Med. Ethics.

[44] Abakumova T.R., Safina A.F., Ziganshina L.E. (2015). Clinical conferences for physicians: Who sets the agenda?. Int. J. Risk Saf. Med..

[45] Fabbri A., Lai A., Grundy Q., Bero L.A. (2018). The Influence of Industry Sponsorship on the Research Agenda: A Scoping Review. Am. J. Public Health.

[46] Moy B., Jagsi R., Gaynor R.B., Ratain M.J. (2015). The impact of industry on oncology research and practice. Am. Soc. Clin. Oncol. Educ. Book.

[47] Rahman M.W., Trivedi N.U., Bach P.B., Mitchell A.P. (2021). Increasing Financial Payments from Industry to Medical Oncologists in the United States, 2014–2017. J. Natl. Compr. Cancer Netw..

[48] Ratain M.J. (2014). Forecasting unanticipated consequences of “The Sunshine Act”: Mostly cloudy. J. Clin. Oncol..

[49] Marshall D.C., Jackson M.E., Hattangadi-Gluth J.A. (2016). Disclosure of Industry Payments to Physicians: An Epidemiologic Analysis of Early Data from the Open Payments Program. Mayo Clin. Proc..

[50] Zhao X., Yang V., Ullah M., Schuweiler M., Zou J., Chen A., Jia S., Ranasinghe P. (2024). The physician payments Sunshine Act and medical oncology: A seven-year financial analysis. Med. Oncol..

[51] Shalowitz D.I., Spillman M.A., Morgan M.A. (2016). Interactions with industry under the Sunshine Act: An example from gynecologic oncology. Am. J. Obstet. Gynecol..

[52] Han E.R., Yeo S., Kim M.J., Lee Y.H., Park K.H., Roh H. (2019). Medical education trends for future physicians in the era of advanced technology and artificial intelligence: An integrative review. BMC Med. Educ..

[53] Rösler W., Altenbuchinger M., Baeßler B., Beissbarth T., Beutel G., Bock R., von Bubnoff N., Eckardt J.N., Foersch S., Loeffler C.M.L. (2023). An overview and a roadmap for artificial intelligence in hematology and oncology. J. Cancer Res. Clin. Oncol..

[54] Duwe G., Mercier D., Kauth V., Moench K., Rajashekar V., Junker M., Dengel A., Haferkamp A., Höfner T. (2025). Development of an artificial intelligence-generated, explainable treatment recommendation system for urothelial carcinoma and renal cell carcinoma to support multidisciplinary cancer conferences. Eur. J. Cancer.

[55] Borus J.F., Alexander E.K., Bierer B.E., Bringhurst F.R., Clark C., Klanica K.E., Stewart E.C., Friedman L.S. (2015). The Education Review Board: A Mechanism for Managing Potential Conflicts of Interest in Medical Education. Acad. Med..

